# Long-term outcomes of surgical repair of isolated coarctation of the aorta in different age groups

**DOI:** 10.1186/s12893-023-02031-5

**Published:** 2023-05-11

**Authors:** Alwaleed Al-Dairy

**Affiliations:** Faculty of Medicine, Damascus University, Children University Hospital, Damascus, Syria

**Keywords:** Congenital heart defect, Coarctation of the aorta, Surgical repair, End-To-End Anastomosis, Recurrent coarctation

## Abstract

**Background:**

Coarctation of the aorta (CoA) is one of the most common congenital heart defects (5–8% of all CHD). Treatment of native CoA may be accomplished surgically, or through an interventional approach. Surgical repair of CoA remains an important option for treatment of aortic coarctation during childhood, although it is mostly performed in neonates and young infants.

**Objectives:**

In this retrospective study, we sought to share the long-term outcomes of different surgical techniques for repair of coarctation of the aorta in different age groups.

**Materials and methods:**

This is a retrospective single-center clinical study that included 228 consecutive patients (age: 1 day- 41years) in whom surgical repair of isolated native coarctation of the aorta was performed with different surgical techniques.

**Results:**

Immediate results were excellent; however, the mortality rate were higher in the infants. Complications rate and incidence of recoarctation, both were comparable between different age groups and different surgical techniques.

**Conclusions:**

Surgical repair of CoA remains an important option for treatment of aortic coarctation in different age groups with low morbidity and mortality. We did not find any significant difference between different surgical techniques regarding the development of recoarctation.

## Introduction

Coarctation of the aorta (CoA) is a congenital narrowing in the aortic isthmus, and represents one of the most common congenital heart defects (CHD) (5–8% of all CHD). Clinical manifestations are variable, ranging from asymptomatic cases diagnosed incidentally in any age group, to cases in which symptoms of congestive heart failure (CHF) may manifest in the neonatal period with ductal dependent systemic circulation [1, 2]. Coarctation may be an isolated lesion, or associated with other CHDs such as ventricular septal defect, bicuspid aortic valve, shone’s complex, hypoplastic left heart physiology, transposition of great arteries, or others [2]. Presentation of isolated CoA with severe congestive CHF in the neonates and young infants is associated with high mortality, and needs immediate medical and surgical management [2]. The introduction of prostaglandins allowed stabilization of the critically ill neonates, and extended the surgical repair to the neonates and very young infants. Diagnosis of CoA may be delayed until childhood or adolescence, and by then, extensive collateral vessels supply blood perfusion distal to the coarctation site [2]. CoA is considered one of the most common causes of secondary hypertension, and without treatment, patients with this anomaly die in the fourth or fifth decade of life from heart failure and cardiovascular sequels of systemic hypertension [1, 3]. Different surgical and interventional techniques for repair have been developed and modified with improved outcomes. Crafoord in 1944 performed the first successful surgical repair of CoA [4–6]. By far, this technique (resection with end-to-end anastomosis (EEA)), and the modified Crafoord technique (extended resection with end-to-end anastomosis (EEEA)), are most commonly used for surgical repair of isolated coarctation, especially in infants and neonates [7]. Subclavian flap aortoplasty (SCAP) is another option for surgical repair of CoA for patients under two years old. When the stenosis is too long for resection and end-to-end anastomosis, Patch aortoplasty (PP) with a prosthetic patch can augment the stenotic area, and this may be useful for older patients [7]. Interposition graft (IPG) may be used more commonly in older patients; however, it had limited role in neonates or infants.

## Materials and methods

In a retrospective study, we reviewed the medical records of the patients who underwent surgical repair of isolated native coarctation of the aorta between January 2000 and December 2019 at our hospital. Baseline demographics, preoperative, intraoperative, and postoperative data were collected from patients’ charts. The study protocol was approved by the local ethics committee in our institution. The patients were regularly followed up in the outpatient clinic (1 month after surgery, then every 3 months), with complete physical examination and transthoracic echocardiography (TTE). The follow-up data were obtained from chart review, with special attention to the development of recoarctation. Patients who had gradients between upper and lower extremities of more than 20 mmHg, and especially those who had “diastolic tail” in color Doppler were further evaluated by computed tomographic angiography or aortography by cardiac catheterization, and luminal narrowing of more than 50% was considered as recoarctation.

### Statistical analysis

Continuous variables were presented as mean ± standard deviation (SD). Qualitative variables were presented as frequency and percentage. Chi Square test, and Fisher’s Exact test were used to compare groups’ means and *P* value < 0.05 was considered statistically significant. All statistical analyses were performed using SPSS 20 for windows (IBM Inc., Somers, NY, USA).

## Results

Our study included 228 patients, of whom 158 were males (69.3%). The patients’ mean age was 80.78 ± 97.13 months (range: 15 days to 41 years). Patients were divided into four age groups infants (≤ 1 year), children (1–12 years), adolescents (> 12 years and ≤ 18 years), and adults (> 18 years) as shown in Table 1.

**Table 1:** The distribution of patients into different age groups

All patients 228 (100%)	Infants 62 (27.2%)	Children 117 (51.3%)	Adolescents 26 (11.4%)	Adults 23 (10.1%)

Children (1–12 years) comprised most of the patients’ cohort (117, 51.3%). The preoperative gradient across the coarctation site as estimated by TTE was 67.2 ± 20.7 mmHg. Different surgical techniques were used: (EEA) in 102 patients (44.7%), (EEEA) in 37 (16.2%), (SCAP) in 34 (14.9%), (PP) in 32 (14%), and (IPG) in 23 (10.1%). The distribution of different surgical techniques according to age groups is summarized in Table 2.

**Table 2:** The distribution of different surgical techniques according to age groups

Technique	EEA	EEEA	PP	SCAP	IPG
Number of patients	102 (44.7%)	37 (16.2%)	32 (14%)	34 (14.9%)	23 (10.1%)
Infants	19 (30.6%)	27 (43.5%)	1 (1.6%)	15 (24.2%)	0 (0%)
Children	70 (59.8%)	10 (8.5%)	14 (12%)	18 (15.4%)	5 (4.3%)
Adolescents	9 (34.6%)	0 (0%)	7 (26.9%)	1 (3.8%)	9 (34.6%)
Adults	4 (17.4%)	0 (0%)	10 (43.5%)	0 (0%)	9 (39%)

Mean aortic clamp time was 34.5 ± 23.11 min (range: 14–80 min). The mean postoperative gradient estimated by TTE was: 20.6 ± 13.5 mmHg. There were 11 in-hospital mortalities (4.8%). Perioperative complications occurred in 13 patients (6%), and included: paraplegia (4 patients, 1.84%), neurologic disorders (3 patients, 1.38%), pneumonia or sepsis (3 patients 1.38%), bleeding requiring surgical re-exploration (2 patient 0.92%), and chylothorax (1 patient 0.46%). The characteristics of in-hospital mortality patients are summarized in Table 3.

**Table 3:** The characteristics of in-hospital mortality patients

Number of Patients	Cause of death	Age group	Surgical technique
4 Patients	Heart failure	Infants	SCAA
		Infants	EEEA
		Infants	EEEA
		Children	EEA
3 Patients	Pneumonia	Infants	SCAA
		Infants	EEA
		Children	EEA
2 Patients	Intensive bleeding	Infants	EEA
		Children	SCAA
1 Patient	Septic shock	Children	IPG
1 Patient	Intracranial hemorrhage	Infants	SCAA

## Follow-up

Mean follow-up duration was 34 ± 11 months (range: 1 to 64 months). There were no late deaths. During follow-up, patients underwent routine physical examination and TTE. Recoarctation developed in 11 patients (5% of the survived patients). The incidence of in-hospital mortality, perioperative complications, and recoarctation according to different surgical techniques, and to different age groups are shown in Tables 4 and 5 respectively.

**Table 4:** The incidence of early mortality, complications, and recoarctation in different surgical techniques

Age group	Infants 62 (27.2%)	Children 117 (51.3%)	Adolescents 26 (11.4%)	Adults 23 (10.1%)	All patients	P value
Mortality	7 (11.3%)	4 (3.4%)	0 (0%)	(0%)	11 (4.8%)	0.07
Complications	3 (5.4%)	8 (7.1%)	2 (7.7%)	0 (0%)	13 (6%)	0.53
Recurrence	1 (1.8%)	10 (8.8%)	0 (0%)	0 (0%)	11 (5%)	0.07

**Table 5:** The incidence of early mortality, complications, and recoarctation in different age groups

Technique	EEA 102 (44.7%)	EEEA 37 (16.2%)	PP 32 (14%)	SCAP 34 (14.9%)	IPG 23 (10.1%)	All patients 228 (100%)	P value
Mortality	4 (3.9%)	2 (5.4%)	0 (0%)	4 (11.8%)	1 (4.3%)	11 (4.8%)	0.12
Complications	5 (5.1%)	3 (8.6%)	1 (3.1%)	3 (10%)	1 (4.5%)	13 (6%)	0.27
Recurrence	8 (8.2%)	0 (0%)	3 (9.4%)	0 (0%)	0 (0%)	11 (5%)	0.11

## Discussion

Treatment of native CoA may be accomplished surgically, or through an interventional approach which has excellent outcomes, and is found to be comparable to surgery in children above one-year-old [8, 9]. However; surgical repair remains an important option for treatment of aortic coarctation during childhood, although it is mostly performed in neonates and young infants nowadays. There is a lack of consensus regarding the preferred technique for surgical repair of CoA repair [10]. Overall perioperative morbidity and mortality rates following CoA surgical repair are low (approximately 2.6% for older children and adults), and mainly determined by age at operation, and presence of associated congenital heart anomalies regardless of the operative technique [11, 12]. In our study, and especially in the first ten years of the study period, interventional approach was not widely used in our hospital, and therefore children (1–12 years) comprised most of the patients’ cohort. The early mortality rate in our study was 4.8%, and there was nearly statistically significant difference between age groups regarding mortality (P value = 0.07), as most mortalities were in the infants’ group. This can be attributed to left ventricular dysfunction that may be present in infants presenting with severe CoA. Moreover, there was not statistically significant difference between different surgical techniques regarding in-hospital mortality. One of the most important issues after surgical repair of CoA is the development of recurrent coarctation. The aortic wall in patients with CoA is histologically abnormal (different smooth muscle and extracellular matrix compared with normal aortic wall), and its affected compliance and distensibility predispose the patient to recurrence of the coarctation after surgical repair [9, 10]. Recurrent coarctation is defined as a residual gradient of more than 20 to 30 mm Hg at the coarctation site. Recoarctation rate has been reported in different studies to be between 5% and 24% [13–18]. It has been reported that surgical repair performed in neonates and young infants has higher rates of recurrence [18–21]. In our study cohort, recoarctation developed in 5% of the survived patients, with nearly statistically significant difference among the different age groups (P value = 0.07), meaning that recoarctation incidence was higher in infants. This is consistent with most articles that study the surgical outcomes of CoA repair, and may be explained by the lack of growth in the anastomosis area. Moreover, we found that the patch plasty technique had the highest incidence of recoarctation (9.4%), and this was consistent with some other reports [22]. Other studies found that recoarctation rates were higher in patients in whom the SCAP technique was used compared to EEA technique [23]. Although we found that PP technique was associated with more recurrence rate; however, P value was not statistically significant (0.07). According to one study; however, the specific surgical repair technique was not important in determining recurrence [24]. Interventional management by balloon angioplasty or endovascular stent placement, is the treatment of choice for most patients with recurrent coarctation [18]. Paraplegia is a rare but disastrous complication of CoA surgical repair. It developed in four of our patients (one infant, and three children). In all these patients, the time of aortic clamping exceeded one hour. It might be useful to apply spinal cord protection methods such as topical cooling. The risk of spinal cord injury nowadays is lower than previous reports [25].

## Conclusion

Surgical repair of CoA remains an important option for treatment of aortic coarctation in different age groups with low morbidity and mortality. We did not find any significant difference between different surgical techniques regarding the development of recoarctation.

## Data Availability

The data that support the findings of this study are available from the corresponding author, [A.A], upon reasonable request.
